# Comparative Transcriptomic Analysis of Two *Brassica napus* Near-Isogenic Lines Reveals a Network of Genes That Influences Seed Oil Accumulation

**DOI:** 10.3389/fpls.2016.01498

**Published:** 2016-09-29

**Authors:** Jingxue Wang, Sanjay K. Singh, Chunfang Du, Chen Li, Jianchun Fan, Sitakanta Pattanaik, Ling Yuan

**Affiliations:** ^1^College of Life Sciences, Shanxi UniversityTaiyuan, China; ^2^Department of Plant and Soil Sciences, University of Kentucky, LexingtonKY, USA; ^3^Cotton Research Institute of Shanxi Academy of Agricultural SciencesYuncheng, China

**Keywords:** *Brassica napus*, lipid biosynthesis, near isogenic lines, seed oil, RNA-sequencing, gene expression, acyl-lipid metabolism

## Abstract

Rapeseed (*Brassica napus*) is an important oil seed crop, providing more than 13% of the world’s supply of edible oils. An in-depth knowledge of the gene network involved in biosynthesis and accumulation of seed oil is critical for the improvement of *B. napus*. Using available genomic and transcriptomic resources, we identified 1,750 acyl-lipid metabolism (ALM) genes that are distributed over 19 chromosomes in the *B*. *napus* genome. *B. rapa* and *B. oleracea*, two diploid progenitors of *B. napus*, contributed almost equally to the ALM genes. Genome collinearity analysis demonstrated that the majority of the ALM genes have arisen due to genome duplication or segmental duplication events. In addition, we profiled the expression patterns of the ALM genes in four different developmental stages. Furthermore, we developed two *B. napus* near isogenic lines (NILs). The high oil NIL, YC13-559, accumulates significantly higher (∼10%) seed oil compared to the other, YC13-554. Comparative gene expression analysis revealed upregulation of lipid biosynthesis-related regulatory genes in YC13-559, including *SHOOTMERISTEMLESS, LEAFY COTYLEDON 1 (LEC1), LEC2, FUSCA3, ABSCISIC ACID INSENSITIVE 3 (ABI3), ABI4, ABI5*, and *WRINKLED1*, as well as structural genes, such as *ACETYL-CoA CARBOXYLASE, ACYL-CoA DIACYLGLYCEROL ACYLTRANSFERASE*, and *LONG*-*CHAIN ACYL-CoA SYNTHETASES*. We observed that several genes related to the phytohormones, gibberellins, jasmonate, and indole acetic acid, were differentially expressed in the NILs. Our findings provide a broad account of the numbers, distribution, and expression profiles of acyl-lipid metabolism genes, as well as gene networks that potentially control oil accumulation in *B*. *napus* seeds. The upregulation of key regulatory and structural genes related to lipid biosynthesis likely plays a major role for the increased seed oil in YC13-559.

## Introduction

In higher plants, cell proliferation, maturation, and desiccation are three key stages of seed development. Seed maturation requires accumulation of storage reserves of carbohydrates, storage proteins, and triacylglycerides (TAG) (Huang, 1992; Goldberg et al., 1994). TAG is high in energy-density, and vital in lipid homeostasis, cellular energy balance, and plant growth and maintenance (Durrett et al., 2008). Seed oils are utilized in human food, raw materials for non-food uses, and high-energy biofuels (Kumar and Sharma, 2015). *Brassica napus* is one of the most important oilseed crops, providing approximately 13% of the world’s supply of vegetable oils (Hajduch et al., 2006). In China, annual *B. napus* production reaches 14 million tons, of which human consumption accounts for more than 6 million tons, approximately 20% of the total edible vegetable oils (Shen and Fu, 2011). *B. napus* seeds contain oil, carbohydrates, and proteins as major storage reserves. The mature seeds contain approximately 45% (w/w) storage oil and 25% (w/w) proteins. The three major seed proteins are cruciferin (40–50%), napin (20%) and a hydrophobic polypeptide associated with the proteinaceous membrane surrounding the storage oil bodies (20%). In addition, about 10% of the storage reserve in *B*. *napus* seeds are soluble sugars (Murphy et al., 1989).

Over the past few decades, significant progress has been made in understanding the lipid biosynthesis using the model plant, *Arabidopsis*, which possesses more than 700 lipid related genes (Beisson et al., 2003). Most of the genes encoding key enzymes in lipid biosynthesis in plants have been isolated and characterized. Previous studies have revealed the importance of fatty acid (FA) metabolism in plant morphology, growth, pollen and seed development, defense, and stress responses (Mou et al., 2000; Kachroo et al., 2003, 2004; Zheng et al., 2005; Zhang et al., 2008; Dong et al., 2009). In most seeds, glycolysis in plastids supplies carbon for FA synthesis; however, green seeds can also use light to generate NADPH and ATP, thus enabling the bypass of glycolysis and an increased metabolic flux (Goffman et al., 2005; Hay and Schwender, 2011). Assembly of TAG occurs in the endoplasmic reticulum (ER) in association with the lipid droplets (LDs), or oil bodies. The plastid FA synthetic pathway determines the acyl chain length and the level of saturation in seed oils. ACETYL-CoA CARBOXYLASE (ACCase) is the first committed enzyme that controls the flux of carbon into FAs. In the plastid, acyl carrier protein (ACP)-linked acyl chains (acyl-ACP) are synthesized by ACCase, FATTY ACID SYNTHASE (FAS), and KETOACYL ACP SYNTHETASE I and II (KASI and II). The saturated acyl-ACP can either be hydrolyzed by the FATTY ACYL-ACP THIOESTERASES B (FATB), or desaturated by acyl-ACP desaturases, and further hydrolyzed by the FATTY ACYL-ACP THIOESTERASES A (FATA) (Li-Beisson et al., 2010). TAG are formed through three sequential acyl-CoA-dependent acylation of the glycerol backbone, catalyzed by enzymes such as ACYL-CoA GLYCEROL-3-ACYLTRANSFERASE (GPAT), ACYL-CoA PHOSPHATIDIC ACID ACYLTRANSFERASE (LPAAT), and ACYL-CoA DIACYLGLYCEROL ACYLTRANSFERASE (DGAT) (Li-Beisson et al., 2010). Although significant progress has been made in isolating structural genes encoding key enzymes, and elucidating their roles in lipid biosynthesis, the regulation of these genes is not well understood. During the past decade, several families of transcription factors (TF) have been reported to be able to enhance seed oil contents. Functionally characterized TFs include SHOOTMERISTEMLESS (STM), LEAFY COTYLEDON1 (LEC1), LEC2, ABSCISIC ACID INSENSITIVE3 (ABI3), FUSCA3 (FUS3) and WRINKLED1 (WRI1) (Mu et al., 2008; Li et al., 2015; Roscoe et al., 2015). STM, a member of the class-I KNOX HOMEODOMAIN-containing proteins, is known to regulate shoot apical meristem architecture in plants. In *Arabidopsis*, altered expression of *STM* affects *in vitro* organogenesis and somatic embryogenesis. Moreover, STM affects somatic embryogenesis by modulating the expression of *LEC1* (Elhiti et al., 2012). LEC1 belongs to the NFY-B type CCAT-box binding TF family; whereas LEC2, ABI3, and FUS3 are plant specific B3-domain TFs. WRI1, a member of the APETALA2/ETHYLENE RESPONSE ELEMENT BINDING PROTEIN (AP2/EREBP) TF family, is a conserved master regulator that controls the genes encoding at least 15 enzymes in FA synthetic and glycolytic pathways, including PYRUVATE DEHYDROGENASE and ACCase (Bates et al., 2013; Ma et al., 2013). ABI3, FUS3, and WRI1 work downstream of LEC1 and LEC2. In addition, WRI1 has been shown to be a direct target of LEC2. *Arabidopsis wri1* mutants accumulate 80% less seed oil (Focks and Benning, 1998). Overexpression of *WRI1* enhances the oil contents in several crop plants, including potato (Hofvander et al., 2016), *B. napus* (Wu et al., 2014; Li et al., 2015), *Camelina sativa* (An and Suh, 2015), and maize (Shen et al., 2010). Recently, the mediator subunit 15 (MED15) has been implicated FA biosynthesis. The mediator is a highly conserved multi-protein complex and an important component of RNA polymerase II-mediated transcription machinery in eukaryotes. *Arabidopsis MED15* overexpression increases FA content in seedlings and mature seeds whereas *MED15* silencing results in reduced FA accumulation. Moreover, MED15 interacts with WRI1 and has been shown to be associated with the promoters of WRI1 target genes (Kim et al., 2016). TFs often mediate the connections between phytohormones and biological processes, such as lipid biosynthesis. Many of these lipid-biosynthesis regulators act downstream of multiple phytohormone pathways (Söderman et al., 2000; Cernac and Benning, 2004; Santos-Mendoza et al., 2008).

*Brassica napus* (genome AACC, 2n = 38) arises from hybridization between the diploids *B. rapa* (Asian cabbage, genome AA, 2n = 20) and *B. oleracea* (Mediterranean cabbage, genome CC, 2n = 18) (Allender and King, 2010). Over the past decade, a growing number of genomic resources for *B. napus* have become available, most noticeably the whole genome sequence of *B. napus* cultivar *Darmor-bzh*, a European winter oilseed (Hilliker et al., 2008; Chalhoub et al., 2014; Wang et al., 2015). Efforts have been made to either identify genomic loci controlling oil contents, or alter the quality and quantity of seed oil of *B. napus* (Katavic et al., 2014; Wu et al., 2014; Elahi et al., 2015, 2016; Li et al., 2015; Liu S. et al., 2016). Niu et al. (2009) have analyzed the FA biosynthesis-related genes using serial analysis of gene expression (SAGE), and concluded that 17–21 days after flowering (DAF) are crucial for the transition of young seeds to sink tissues (Niu et al., 2009). Transcriptome analysis of developing seeds from four different plant species, *Ricinus communis, B. napus, Euonymus alatus* and *Tropaeolum majus*, which differ in their oil storage tissues, as well as TAG structures and contents, has revealed both conserved and distinct species-specific expression patterns for the genes involved in lipid biosynthesis (Troncoso-Ponce et al., 2011). Recently, a comparative mapping of *Arabidopsis* lipid-related orthologous genes in *B. napus* has also been reported (Subramaniam et al., 2011).

As a close relative to *Arabidopsis, B. napus* is considered an ideal crop plant for translating information from the model species (Snowdon and Friedt, 2004). However, frequent duplication and rearrangement events in the *B. napus* genome preclude the establishment of a simple one-to-one relationship between *B. napus* and *Arabidopsis* (Parkin et al., 2005). Although the biochemical pathway for *de novo* lipid biosynthesis is well understood, much less is known about how qualitative and quantitative lipid productions are controlled during seed development of oil seed crops, such as *B. napus*. Advancements in high throughput technologies have resulted in surge of various -omic data addressing different aspects of plant growth and development. Microarray and next-generation sequencing (NGS) technologies have revolutionized our ability to monitor and analyze the global transcriptomic changes of plants in response to developmental and environmental cues. Genome-wide transcriptomic analyses of genetic variants can lead to identification of a set of genes that contribute to phenotypic changes. An insightful approach to understand the multi-step TAG biosynthetic pathway, and its complex regulatory network, is to study plant near isogenic lines (NILs) with near identical genetic background but different levels of TAG accumulation. Transcriptomic analysis of such NILs can potentially yield distinctive gene expression profiles that allow the identification of critical genes involved in TAG biosynthesis and candidate target genes for further characterization. For this purpose, we have developed a pair of *B. napus* NILs that vary in seed oil content by ∼10%. We identified differentially expressed genes (DEGs) of the two NILs. Our results suggest that changes in expression of the genes related to phyotohormones and lipid-regulating TF likely resulted in increased expression of FA and TAG pathway genes in the high oil-accumulating NIL, YC13-559.

## Materials and Methods

### Plant Materials

The parental donor of the NILs was a high-oil *B. napus* variety, YN171, developed by the Nanjing Agricultural Research Institute, China. Breeding in Shanxi province to select regional agronomic performance, such as growth, yield, and oil contents, has developed several *B. napus* lines. The NILs were developed by conventional method of repeated back-crossing (BC) of YN171 with locally domesticated recipient for up to five generations. BC individuals were then selfed and a number of lines selected from each crossing stream. YC13-554 and -559 are two individual NILs having genetic similarity coefficient of 0.94 as determined by SSR analysis. FA analysis also showed that YC13-559 accumulated significantly higher oil (∼10%) than YC13-554 (Liu Z. et al., 2016). This pair of NILs were selected for three consecutive years of field trial for agronomic characteristics and oil accumulation.

Near isogenic lines, YC13-554 and YC13-559, were grown in experimental fields in Yuncheng city, Shanxi, China, in the 2014 growing season. Following artificial pollination, siliques in the middle of main florescence were date-tagged. Immature seeds were separated from siliques at 19 days after pollination (DAP) as described previously (Niu et al., 2009), frozen immediately in liquid nitrogen, and stored at -80°C until total RNA isolation.

### RNA Isolation and Illumina Sequencing

Total RNA were isolated from 2 g of immature seeds collected at 19 DAP using the RNeasy Plant Mini Kit (Qiagen, Chatsworth, CA, USA) following manufacturer’s instructions. RNA quantity was determined using a NanoDrop ND-1000 spectrophotometer (NanoDrop Technologies, Wilmington, DE, USA). Quality of RNA samples were determined using an Agilent 2100 Bioanalyzer (Agilent Technologies, Palo Alto, CA, USA). RNA samples with RNA integrity number (RIN) above 8 were used for library preparation. cDNA libraries were prepared using the TruSeq RNA Sample Prep Kit (Illumina, San Diego, CA, USA) according to the manufacturer’s protocol. The libraries were then pooled together and sequenced on an Illumina HiSeq 2000 (2X 101bp).

### Gene Expression Quantification

Raw Illumina sequence reads were processed using the prinseq-lite-0.20.4 (Schmieder and Edwards, 2011) for removal of low-quality reads (Singh et al., 2015). Subsequently, pre-processed reads were assessed for quality control with FastQC (version 0.11.3; Babraham Bioinformatics, Cambridge, UK). Read mapping was performed by Bowtie2 (Langmead and Salzberg, 2012) using reference sequence downloaded from the *B. napus* genome database (Chalhoub et al., 2014). Differential gene expression analysis was carried out using the DESeq2 Bioconductor package in R (Love et al., 2014). DEGs were identified using two criteria: (a) log2 fold-change ≥ 1 and (b) an adjusted *p*-value less than 0.05.

### Gene Ontology Analysis

Corresponding *Arabidopsis thaliana* orthologs for all DEGs were determined by the reciprocal best hits method. A list of *Arabidopsis* orthologs was uploaded to BiNGO plug-in (Maere et al., 2005) of Cytoscape (Shannon et al., 2003) for gene ontology analysis. Enrichment analysis was based on a hypergeometric test. *P*-values were adjusted using Benjamini–Hochberg’s FDR; only FDR < 0.05 was considered significant. For pathway analysis, a MapMan mapping file was specifically generated for *B. napus* genes by the Mercator tool, which bins all genes according to hierarchical ontologies after searching a variety of databases. MapMan v.3.5.1 (Thimm et al., 2004) was used to visualize the DEGs. In this study, the MCScanX tool kit (Wang et al., 2012a) with default parameter was used to identify genome collinearity. The Protein ANalysis THrough Evolutionary Relationships (PANTHER) Classification System and analysis tools were used to categorize DEGs by protein class (Mi et al., 2016).

### Identification and Expression Analysis of Acyl-lipid Metabolism Genes of *B. napus*

In order to identify genes related to acyl-lipid metabolism, *B. napus* gene sequences were analyzed against a list of acyl-lipid metabolism genes obtained from the “*Arabidopsis* Acyl-Lipid Metabolism” website (ARALIP)^1^. Locally installed MultiLoc2 tool was used for prediction of sub-cellular localization of proteins (Blum et al., 2009).

To analyze the temporal expression of acyl-lipid metabolism genes in sequential stages of seed filling of *B. napus*, RNA-seq data of seeds at four developmental stages (2, 4, 6, and 8 weeks after pollination), and microarray data of eight developmental stages (10, 15, 20, 25, 30, 35, 40, and 45 days), were obtained from the sequence read archive database (accession number SRP069360) and Gene Expression Omnibus (accession number GSE43918), respectively (Leinonen et al., 2011; Barrett et al., 2013). Unsupervised hierarchical clustering was performed using *hclust* function, and clusters were then extracted using the *cuttree* function in R base package^2^. The heat-map corresponding to the hierarchical clustering was generated with the *heatmap.2* function of the R packages *gplots*^3^.

### Quantitative Real-Time PCR

RNA isolated from immature seeds of YC13-554 and YC13-559 were reverse-transcribed using the Superscript III Reverse Transcriptase (Invitrogen, USA), following the manufacturer’s instructions. Quantitative real-time PCR (qRT-PCR) was performed as described previously (Pattanaik et al., 2010). All PCR reactions were performed in triplicate and repeated two times. The comparative cycle threshold (Ct) method (bulletin no. 2; Applied Biosystems)^4^ was used to measure transcript levels. *B. napus* actin (GenBank accession number AF111812) was used as a reference gene (Niu et al., 2009). The relative expression of each gene is presented as the ratio of that in YC13-559 and YC13-554. The normalized expression in YC13-559 was divided by the normalized expression in YC13-554 and log2 transformed. The data represent the mean values ± SD of three replicates. Primer sequences for the genes have been listed in Supplementary Table S5.

## Results and Discussion

### The Near Isogenic Lines YC13-559 and YC13-554 Differ Significantly in Oil Content

Near isogenic lines, with near identical genetic background but varying mainly in the alleles responsible for a trait of interest, are useful in identification of target genes and pathways. Starting in 2009, we developed two *B. napus* NILs, YC13-554 and YC13-559, through repeated backcrossing of a high-yielding production line, YN171 with the recipient line. The two NILs share a genetic similarity coefficient of 0.94 based on SSR marker analysis. Agronomic trait analysis of the NILs showed several major differences, including 1000-seed weight and seed oil content. While plant height, primary branch height, length of main inflorescence, and number of pods per main inflorescence, were indistinguishable, YC13-559 accumulated significantly higher (∼10%) seed oil than YC13-554. The 1000-seed weight, which negatively correlates with seed oil content (Tang et al., 1997), is 30% less in YC13-559 compared to YC13-554. High oil accumulation is inversely correlated with protein content in seeds. As no significant seed size difference between the two NILs was observed, we speculate that a decrease of proteins or other metabolites led to the reduced seed weight. The results of three consecutive years of field trial (2012–2014) demonstrated that oil accumulation was higher in YC13-559 compared to YC13-554. It is important to note that the data generated in this study were based on field trial results. In field production, even a small percentage increase in seed oil translates into significant crop values (Lardizabal et al., 2008). The two NILs are ideal materials for the investigation of genetic and gene regulatory variations that contribute to TAG accumulation.

### Analysis of Acyl-lipid-Metabolism Gene Expression during *B. napus* Seed Development Reveals Genetic Complexity and Critical Timing for TAG Biosynthesis

**FIGURE 1:**
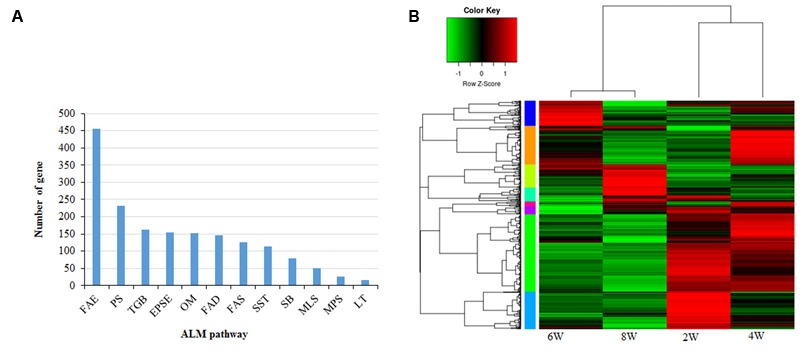
**Acyl-lipid-metabolism (ALM) genes in *Brassica napus***(A)** Identification and distribution of ALM genes in *B. napus* into twelve different functional categories and number of genes in each category is shown **(B)** ALM genes were categorized into 8 distinct clusters based on their expression pattern.** Heat map shows the relative expression of all groups. Clusters are color-coded by row sidebars: light orange (cluster 1), green–yellow (cluster 2), green (cluster 3), cyan (cluster 4), aqua (cluster 5); blue (cluster 6); purple (cluster 7) and pink (cluster 8). FAE, Fatty Acid Elongation; PS, Phospholipid Signaling, TGB, Triacylglycerol Biosynthesis; EPSE, Eukaryotic Phospholipid Synthesis and Editing; OM, Oxylipin Metabolism; FAD, Fatty Acid Degradation; FAS, Fatty Acid Synthesis; SST, Suberin Synthesis Transport; SB, Sphingolipid Biosynthesis; MLS, Mitochondrial Lipopolysaccharide Synthesis; MPS, Mitochondrial Phospholipid Synthesis; LT, Lipid Trafficking; 2W, 2 weeks; 4W, 4 weeks; 6W, 6 weeks and 8W, 8 weeks.

The amphidiploid *B. napus* shows significantly greater genome-level divergence compared to its close relative, *Arabidopsis*. We sought to clarify the number and distribution of acyl-lipid-metabolism (ALM) genes in *B. napus*. Based on homologies to 771 *Arabidopsis* ALM genes, we identified a total of 1750 lipid-related genes in *B. napus* (Supplementary Table S1). *B. rapa* and *B. oleracea*, two diploid parents of *B. napus*, contributed almost equally to the ALM genes; 881 and 863 genes were derived from the AA and CC genomes, respectively (Supplementary Table S1). *B. napus* ALM genes were found to be present in over 19 chromosomes, with chromosomes A03 and C03 harboring the largest numbers of ALM genes (126 and 122, respectively), while chromosomes A06 and C06 contain the least 70 and 68, respectively. **Figure 1A** summarizes the distribution of these genes into 12 different functional categories. The most abundant ALM genes were found to function in FA elongation, followed by phospholipid signaling and TAG biosynthesis. The maximum numbers of ALM proteins (∼37%) were predicted to be distributed in the cytoplasm, followed by organelles involved in ALM, such as the ER (∼18%) and chloroplasts (∼12%) (Supplementary Table S1). Whole-genome, segmental, and tandem gene duplications are common mechanisms for the generation of evolutionary novel functions (Wang et al., 2012b). In this study, we used the MCScanX toolkit (Wang et al., 2012a), with default parameters to detect collinear genomic regions and classify duplicated ALM genes according to their most likely mode of generation. The ALM genes, distributed on all nineteen chromosome pairs, were subjected to substantial duplication events (Supplementary Table S1). Among the duplicated genes, 1499 (85.6%) were predicted to have originated from whole genome duplication or segmental duplication events (Supplementary Table S1). The significantly higher numbers of ALM genes found in *B. napus* compared to *Arabidopsis*, are most likely due to frequent duplication events.

Developing seeds accumulate TAGs either in embryonic tissues (e.g., *B. napus* and soybean), or in the endosperm (e.g., castor bean). Analyzing the specific gene expression patterns during seed development will aid in understanding the mechanism of seed oil accumulation. We examined the temporal changes in ALM gene expression during *B. napus* seed filling by analyzing RNA-seq data. Expression profiles of ALM genes during different stages of seed development were studied using publicly available RNA-seq datasets deposited in sequence read archive (SRA) database (accession number SRP069360). These datasets contain the expression data for four seed developmental stages, i.e., 2, 4, 6, and 8 weeks after pollination (WAP). To avoid background noise, ALM genes having expression levels equal to, or more than 1, read per kilobase per million (RPKM) in at least two samples, were selected for expression analysis (Topa and Honkela, 2016) (Supplementary Table S2). A total of 1205 genes thus generated from four developmental stages were grouped into 8 different clusters based on their expression patterns (**Figure 1B**; Supplementary Figure S1). Genes in cluster 1 (*n* = 203) and 3 (*n* = 408), which constitute ∼50% of all analyzed genes, were most highly expressed during week 4 of seed development (Supplementary Figure S1). Members of these two clusters are mainly involved in FA synthesis, FA elongation, and phospholipid signaling. As seeds aged, cluster 2 (*n* = 123) gene expression increased continuously, while cluster 5 (*n* = 198) gene expression decreased. Cluster 2 was overrepresented by genes involved in TAG biosynthesis, whereas cluster 5 genes are mostly related to FA elongation, phospholipid signaling, and TAG degradation. The expression of genes related to FA-elongation, TAG synthesis, and degradation peaked in week 4 (Supplementary Figure S2). Between weeks 6 and 8, the expression of most lipid related genes were gradually reduced. Previous studies on *B. napus* seed filling (Hajduch et al., 2006; Jolivet et al., 2011) show that seed weight, FA accumulation, and protein production increase continuously during the first 5 weeks after flowering. Seed weight and FA synthesis remain unchanged between weeks 4 and 5, while protein production continues. Our results are in good agreement with the reported characteristics of *B. napus* seed filling, showing the timing of ALM gene expression is slightly ahead of lipid accumulation.

To further verify our data, we analyzed a microarray dataset, obtained from Gene Expression Omnibus (accession number GSE43918), that includes *B. napus* tissues from week 2–6 of seed development (Huang et al., 2013). A total of 597 probes that mapped uniquely to the ALM genes, were included in this analysis (Supplementary Table S3). As shown in Supplementary Figure S3A, *B. napus* ALM genes formed three clusters, corresponding to week 2 (10 and 15 days), week 3–4 (20, 25, and 30 days), and week 5–6 (35, 40, and 45 days), of seed filling. Based on expression patterns during seed development, all probes were further classified into 16 different clusters. Similar to RNA-seq data, and a previous report (Niu et al., 2009), we observed that approximately 50% of analyzed probes were expressed the highest at 3–4 weeks (Supplementary Figure S3B). Seed LDs comprise a TAG core surrounded by a monolayer of phospholipids embedded with proteins such as caleosin and oleosin. Consistent with a report by Jolivet et al. (2011), we found that the expression of caleosin- and oleosin-family genes increased as seeds aged. Several TF genes that are known to be involved in FA biosynthesis and TAG accumulation, including *WRI1, FUS3*, and *ABI3*, have their highest expression in 3–4 weeks. Collectively, both RNA-seq and microarray analysis revealed that weeks 3–4 during seed development is critical for TAG biosynthesis in *B. napus* as expression of genes encoding key enzymes and transcriptional regulators peak at this stage.

### RNA-seq Identifies Differentially Expressed Genes in the NILs

**FIGURE 2:**
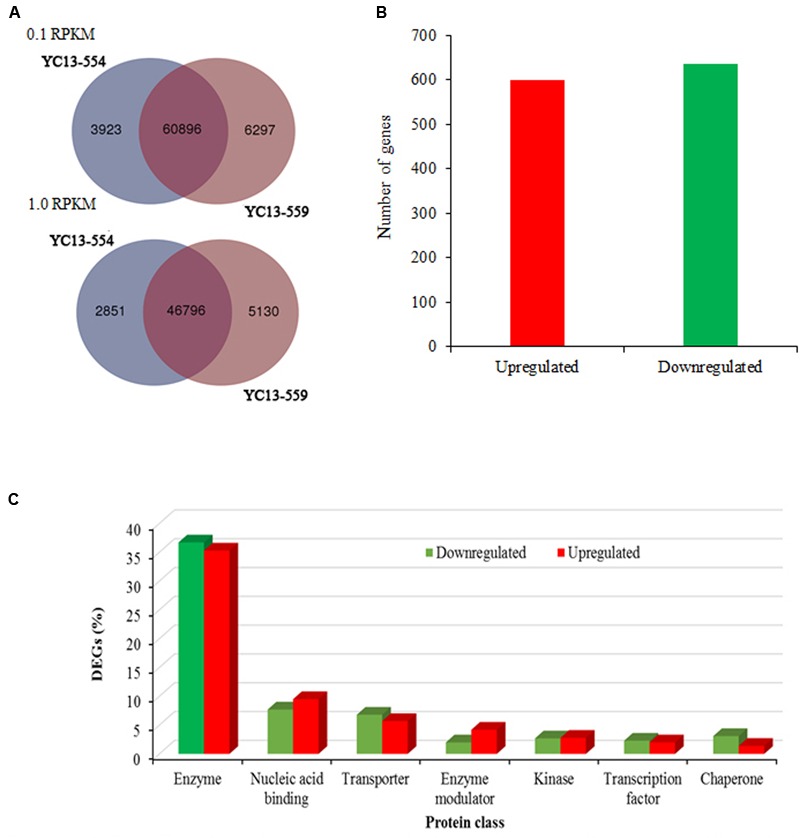
**Differential expression and classification of transcripts. (A)** Venn diagrams showing overlap of expressed genes having at least 0.1 reads per kilobase of transcript per million mapped reads (RPKM) and 1.0 RPKM expression value in near- isogenic lines YC13-554 and YC13-559. **(B)** Number of upregulated and downregulated genes. **(C)** Protein classification of the DEGs (*n* = 1234 genes; 635 upregulated and 599 downregulated) using PANTHER (Protein Analysis Through Evolutionary Relationships). Protein classes encompassing at least 2% of the upregulated or downregulated genes are shown.

Illumina transcriptome sequencing was performed for libraries prepared from the two NILs. After the quality control, RNA-seq generated approximately 23.5 and 22.6 million pair-end reads (100-bp in size), as well as 7.9 and 7.5 million singletons for YC13-554 and YC13-559, respectively (**Table 1**). On an average, 86% reads were mapped to the reference genome using the software, Bowtie2 (Langmead and Salzberg, 2012). Based on RPKM, we found close overlap between the two NILs. When 0.1 RPKM was set as the threshold, 60,896 common genes were found to express in both NILs. When the threshold was increased to 1 RPKM, 46,797 common genes were found (**Figure 2A**). We identified 1234 candidate DEGs (twofold change) between the two NILs (**Figure 2B**, Supplementary Table S4). In YC13-559, 635 genes were upregulated, while 599 genes were downregulated compared to YC13-554. The upregulated and downregulated DEGs were classified into different class of proteins using PANTHER. More than 35% of DEGs belonged to enzyme class of proteins. Nucleic acid binding proteins and transporters were two other abundant class of proteins in DEGs (**Figure 2C**).

**Table 1:** Summary of transcriptome data.

	YC13-554		YC13-559	
	R1	R2	R1	R2
Raw reads	39,101,234	39,101,234	37,673,205	37,673,205
Total number of paired-end reads	23,529,581	23,529,581	22,688,191	22,688,191
Total number of unique paired-end reads	11,657,929	11,915,210	11,895,558	11,683,396
Total number of singletons	4,405,652	3,528,907	4,391,647	3,295,361
Total number of unique singletons	3,124,713	2,587,097	3,220,975	2,508,251

### Quantitative RT-PCR of Selected ALM Genes Validates the RNA-seq Results

**FIGURE 3:**
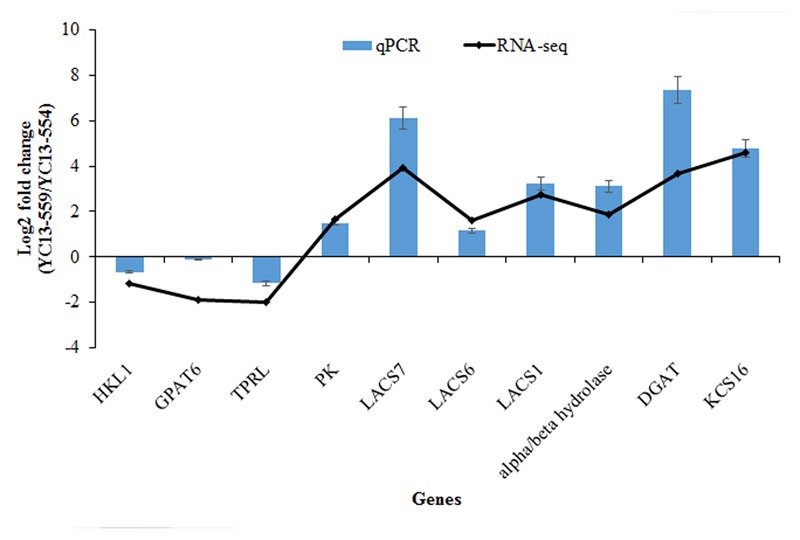
**Validation of RNA-seq results using quantitative real-time polymerase chain reaction (qRT-PCR).** The relative abundance of each gene is presented as the ratio of YC13-559 and YC13-554 lines. *B*. *napus actin* was used as an internal control for normalization. The normalized expression (2^-ΔCT^) in YC13-559 sample was divided by the normalized expression in YC13-554 and log2 transformed. The data represent the mean values ± SD of three replicates. *HKL1, hexokinase-like 1; GPAT6, glycerol-3-phosphate acyltransferase 6; TPRL, tetratricopeptide repeat (TPR)-like superfamily; protein PK, pyruvate kinase family protein; LACS7, long-chain acyl-CoA synthetase 7; LACS6, long-chain acyl-CoA synthetase 6; LACS1, long-chain acyl-CoA synthetase 1; alpha/beta-Hydrolases, alpha/beta-Hydrolases superfamily protein; DGAT1, acyl-CoA:diacylglycerol acyltransferase 1; KCS16, 3-ketoacyl-CoA synthase 16*.

We identified a number of FA biosynthesis- and accumulation-related genes whose expression changed significantly in YC13-559 compared to YC13-554 and chose a set of 10 genes (7 upregulated and 3 downregulated) from that list to validate their expression by qRT-PCR (**Figure 3**; Supplementary Table S5). In addition to the 3 downregulated genes, 7 ALM genes, including *pyruvate kinase (PK), long-chain acyl-CoA synthetase1, 6, and 7 (LACS1, 6, and 7), alpha/beta-Hydrolases superfamily protein (alpha/beta hydrolase), DGAT1*, and *3-ketoacyl-CoA synthase 16 (KCS16)*, were upregulated. The qRT-PCR results were in agreement with the RNA-seq data, confirming the quality and accuracy of our RNA-seq experiment.

### Gene Ontology and MapMan Functionally Classify the Differentially Expressed Genes

**FIGURE 4:**
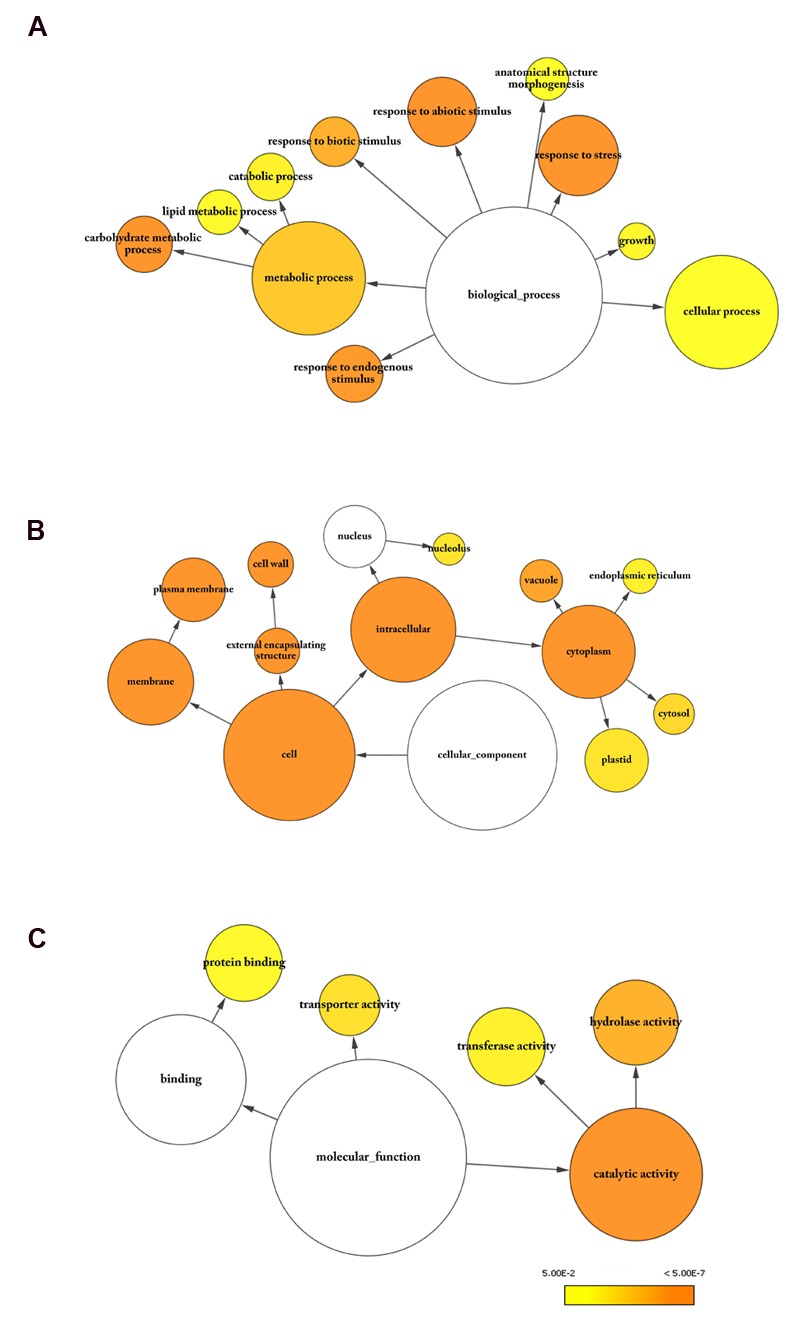
**Gene Ontology (GO) analysis of DEGs.** GO analysis was carried out using BinGO (Maere et al., 2005). Enriched categories for **(A)** biological process, **(B)** cellular component, and **(C)** molecular function are shown. Goslim_Plants categories with significant enrichment in the dataset were highlighted in color. The size of the node is proportional to the number of molecules within the group, and the color of the node represents the significance of enrichment. Benjamini and Hochberg *p*-value legend is indicated below.

Gene Ontology (GO) annotation is a major tool for gene enrichment analysis of genome-scale experiments. The enrichment analysis of the transcriptomic data from the two NILs, according to three major GO terms, namely the biological process, cellular component and molecular function, and, is given in **Figure 4**. The GO term, “biological process,” includes the majority of genes involved in metabolic process (GO: 0008152), cellular process (GO: 0009987), and response to stress (GO: 0006950) (**Figure 4A**). Lipid metabolic process (GO: 0006629) and carbohydrate metabolic process (GO: 0005975) were two most affected metabolic processes. Lipid metabolism is closely connected to that of carbohydrates which are regularly metabolized to form acetyl-CoA, a precursor for FA biosynthesis. The metabolism process was overrepresented by children terms, including lipid biosynthesis (GO: 0006629), monocarboxylic acid biosynthesis (GO: 0072330), and oxylipin metabolism (GO: 0031407). Several genes related to JA and oxylipin metabolism, such as *LIPOXYGENASE 2* (*LOX2*), *LOX3, JASMONIC ACID CARBOXYL METHYLTRANSFERASE (JMT)*, and *JASMONATE RESISTANT 1 (JAR1)*, were found to be differentially expressed in our transcriptome data. JA and FA synthesis are well connected as the acyl-CoA pool generated from FA biosynthetic pathway is used for JA synthesis (Fu et al., 2015). The DEGs in the category “cellular components” were predicted to localize mostly in cytoplasm or plasma- and cell-membranes (**Figure 4B**). Notably, cell organelles, such as ER and plastid, which are known sites of FA and TAG biosynthesis, are also enriched in our dataset. Within the category “molecular function,” the most abundant GO term is catalytic activity (GO: 0003824), followed by protein binding (GO: 0005515) (**Figure 4C**). The majority of catalytic activities are associated with hydrolases and transferases.

**FIGURE 5:**
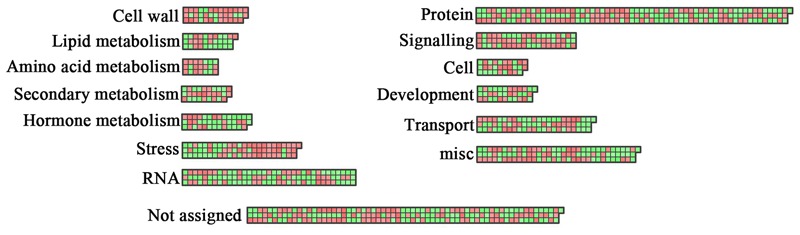
**Functional analysis of DEGs using MapMan.** Overview of the distribution of DEGs in different functional groups as identified by MapMan (Thimm et al., 2004) analysis. Red boxes correspond to up-regulated genes and green boxes to down-regulated genes.

As a complementary approach to GO term enrichment analysis, we explored the putative functions of the DEGs using MapMan (Thimm et al., 2004), which allows the visualization of varietal specific changes in different metabolic processes (**Figure 5**). Each gene in MapMan is initially organized in bins rather than as pathways, allowing the genes to be assigned into a pathway even when their functions are only tentatively predicted. **Figure 5** shows the mapping of the DEGs to overall metabolism, cellular responses, and regulation. Consistent with the GO analysis, the DEGs were enriched in similar functional categories and pathways by MapMan. Although a significant fraction of DEGs could not be assigned to specific functional categories, a large number of genes were assigned to lipid metabolism, cell wall, protein, signaling, and transport. Notably, many genes were linked to stress, consistent with the involvement of lipid related genes in response to both biotic (Shah, 2005) and abiotic stress (Golldack et al., 2014; Higashi et al., 2015; Hou et al., 2016). An additional bin comprises genes involved in biosynthesis of secondary metabolites, such as flavonoids and phenylpropanoids. Another significantly affected category involves genes related to phytohormone biosynthesis and signaling. A total of 41 genes related to JA, ethylene, abscisic acid, gibberellins (GAs), auxin, and brassinosteroid were found to be differentially expressed. Previously, altered FA productions have been reported in *Arabidopsis* mutants deficient in JA and auxin metabolism (Niu et al., 2009). GAs play crucial roles in many aspects of plant life cycles, from seed germination and development to reproduction. GA signal perception and transduction are controlled by the GA receptor GIBBERELLIN INSENSITIVE DWARF (GID) and DELLA repressor proteins (Daviere and Achard, 2013). In many plant species, GA biosynthesis increases in the early stages of seed development, continues to seed maturity, and begins to decrease at the onset of dormancy. GA_3_ is also involved in the regulation of FA biosynthesis in *Arabidopsis*. Loss of DELLA function or exogenous application of GA_3_ affect the expression of key regulatory genes, including *LEC1, LEC2, FUS3, ABI3* and *WRI1*, as well as their targets in *Arabidopsis* (Chen et al., 2012). A cytochrome p450, *ent*-kaurenoic acid oxidase (KAO), catalyzes the three-step oxidation of *ent*-kaurenoic acid to GA_12_, the precursor of all GAs, thereby determining the endogenous GA levels in plants (Regnault et al., 2014). In our data set, the expression of a *KAO1* homolog was significantly higher in YC13-559 compared to YC13-554. The higher expression of *KAO1* likely resulted in the increase of endogenous GA level, and was probably, at least in part, responsible for increased expression of the regulatory and structural genes in YC13-559 FA biosynthetic pathway. Inactivation of phytohormones is a mechanism to maintain hormone homeostasis in plants. In *Arabidopsis*, two members of the SABATH family methyltransferases, GIBBERELLIN METHYLTRANSFERASE (GAMT) 1 and GAMT2, mediate the transfer of methyl groups to GA, resulting in the biologically inactive methyl esters. Overexpression of *GAMT1* or *GAMT2* in *Arabidopsis*, petunia, and tobacco lead to GA deficiency and the resulting transgenic plants are dwarf with reduced fertility (Varbanova et al., 2007). In our data set, the expression of a *GAMT* homolog was higher in YC13-559 compared to YC13-554. It appears that the biosynthesis and catabolism of GA, involving activation and deactivation of the GA, are affected more significantly in YC13-559 than in YC13-554.

Jasmonic acid is essential for a number of biological processes in plants, including growth, development, and response to wounding. Long-chain FAs serve as the precursors of JA biosynthesis in plants. CORONATINE INSENSITIVE 1 (COI1) plays a key role in JA signal perception and transduction. The *Arabidopsis* loss-of-function mutant, *coi1*, exhibits altered FA biosynthesis and composition in seeds (Niu et al., 2009). On the other hand, mutations in key FA biosynthetic genes have been shown to affect JA signaling. Mutation in the *Arabidopsis ssi2* locus, that encodes the plastidic stearoyl–ACP desaturase, compromises JA responses (Kachroo et al., 2003; Nandi et al., 2003), suggesting the existence of a feedback regulatory loop between the JA signaling and FA biosynthesis pathways. A number of genes encoding enzymes for JA biosynthesis and conversion of JA to methyl jasmonate (MeJA) were differentially expressed in our data set. Increased expression of genes encoding *LOX2*, that oxidizes ś-linolenic acid, and *JMT*, that converses JA to MeJA, were observed in YC13-559 relative to YC13-554.

The category ‘RNA’ includes the genes related to RNA metabolism and TFs (Supplementary Table S6). A total of 57 TFs in this category can be further classified into 28 families that are overrepresented by MYB, WRKY, and homeobox TFs. FA biosynthesis is a rigorous process in which transcriptional regulation is no doubt vital; however, to date only very few TFs have been identified to be responsible for FA biosynthesis and storage. We thus analyzed the co-expression of these TFs and the FA biosynthetic genes. A total of 1262 genes (1205 ALM and 57 TF genes), were categorized into 7 clusters (Supplementary Figure S4A). Clusters 3 and 4 were the largest clusters containing approximately 50% of the identified TFs. The members of cluster 3 were found to have highest expression at early stage (2-week) of seed filling, while the members of cluster 4 maximally expressed during week 4 (Supplementary Figure S4B). Cluster 3 and 4 are rich in MYB, AP2/ERF and Dof TFs. Some members of these TF families are known to be involved in direct or indirect regulation of ALM genes. For example, overexpression of *GmDof4* or *GmDof11* from *Glycine max* enhance lipid content in transgenic *Arabidopsis* (Wang et al., 2007).

### Key Regulatory Genes in FA and TAG Biosynthetic Pathways are Upregulated in YC13-559

**FIGURE 6:**
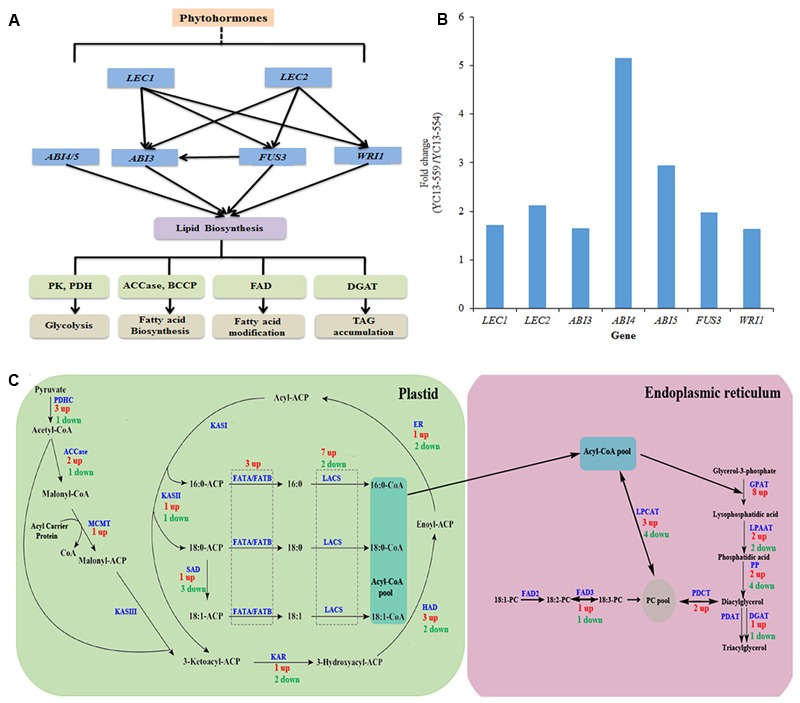
**Overview of gene network involved in seed oil biosynthesis in *B*. *napus***(A)**.** A simplified scheme of transcription factors (TF) network involved in regulation seed oil biosynthesis *B*. *napus* **(B)** Relative expression levels of different TF genes in YC13-559. TFs whose expression changed more than 1.5-fold in YC13-559 relative to YC13-554 are shown. The RPKM values for multiple copies of same TF were summed. **(C)** Overview of *de novo* fatty acid (FA) and triacylglycerol (TAG) biosynthesis pathways. Genes whose expression changed more than 1.5-fold in YC13-559 relative to YC13-554 were included in this study. Genes were mapped onto *de novo* FA and TAG biosynthesis pathway. Numbers refers to the numbers of genes, and “up” refers to up-regulation in YC13-559 line compare to YC13-554 line, and “down” to down-regulation. Same enzyme catalyze several steps are highlighted with dotted rectangle. Lipid substrates are abbreviated: 16:0, palmitic acid; 18:0, stearic acid. PDHC, pyruvate dehydrogenase complex; ACCase, acetyl-CoA carboxylase; MCMT, malonyl-CoA : ACP malonyltransferase; ACP, acyl carrier protein; KASI/II/III, ketoacyl-ACP synthase I/II/III; KAR, ketoacyl-ACP reductase; HAD, hydroxyacyl-ACP dehydrase; ER, enoyl-ACP reductase; FATA/B, fatty acyl-ACP thioesterase A/B; SAD, stearoyl-ACP desaturase; LACS, long-chain acyl-CoA synthetase; GPAT, glycerol-3-phosphate acyltransferase; LPAAT, 1-acylglycerol-3-phosphate acyltransferase, PP, phosphatidate phosphatase; PDCT, Phosphatidylcholine:diacylglycerol cholinephosphotransferase; PDAT, phospholipid :diacylglycerol acyltransferase; DGAT, acyl-CoA : diacylglycerol acyltransferase, LPCAT, lysophospholipid acyltransferase; FAD2/3, fatty acid desaturase.

Expression of the structural genes in FA and TAG biosynthetic pathways are regulated by a number of TFs, including STM, LEC1, LEC2, ABI3, FUS3, and WRI1 (**Figure 6A**). Recently, ABI4, an AP2/ERF TF, and ABI5, a bZIP TF, have also been implicated in TAG biosynthesis in *Arabidopsis* (Yang et al., 2011; Kong et al., 2013). Accumulating evidence has begun to uncover the gene regulatory network underlying lipid biosynthesis in plants. LEC1 and LEC2 likely act downstream of *STM*, but upstream of *ABI3* and *FUS3*. ABI3 and FUS3 are known to regulate each other (**Figure 6A**) (Baud et al., 2007; Mu et al., 2008; Santos-Mendoza et al., 2008; Maeo et al., 2009). Expression of *STM, LEC1, LEC2, ABI3*, and *FUS3*, were moderately higher in YC13-559 compared to YC13-554. The *B. napus* genome contains two *LEC1* and four *LEC2* homologs of *Arabidopsis*. Expression of two *LEC1* and three *LEC2* homologs were 1.7–2.5 fold higher in YC13-559 relative to YC13-554. *B. napus* contains four FUS3 homologs of *Arabidopsis* and expression of one was higher in YC13-559 compared to YC13-554. In *Arabidopsis, WRI1* is directly controlled by LEC1 (Mu et al., 2008) and LEC2 (Baud et al., 2007). We therefore analyzed the expression of the genes encoding WRI1 in the two NILs. *B. napus* has 3 *Arabidopsis WRI1* homologs. Expression of all three homologs were 1.5–1.8 fold higher in YC13-559 compared to YC13-554. WRI1 regulates expression of the late step genes in the glycolic and FA biosynthetic pathways. Enhanced expression of *WRI1* homologs in YC13-559 correlated with expression of the genes involved in seed oil biosynthesis, such as *PK, PDHC*, and *LACS*, known targets of WRI1 (Pouvreau et al., 2011). ABI4 and ABI5 repress lipid breakdown by increasing expression of *DGAT1*, a rate-limiting gene in TAG biosynthesis (Yang et al., 2011; Kong et al., 2013). The expression of *ABI4* and *ABI5* were 5- and 3-fold higher, respectively, in YC13-559 relative to YC13-554 (**Figure 6B**).

### A Number of Key Structural Genes in the FA and TAG Biosynthetic Pathways Are Upregulated in YC13-559

Biosynthesis of TAG involves three major steps in two sub-cellular compartments (**Figure 6C**). The first step is *de novo* FA synthesis in the plastid, mainly by the FA synthase complex (FAS) using acetyl-CoA as starting substrate. Subsequently, TAG is derived from FA and glycerol backbone in the ER. Finally, TAG is stored, by association with LDs, in the cytoplasm (Li-Beisson et al., 2010; Bates et al., 2013). PK catalyzes the conversion of phosphoenol pyruvate (PEP) to pyruvate, which is translocated to the plastid via the BILE ACID: SODIUM SYMPORTER FAMILY PROTEIN 2 (BASS2) transporter, and acts as an initial precursor for FA biosynthesis (Furumoto et al., 2011). Expression of *PK* and *BASS2* were higher (1.6–10 fold) in YC13-559 compared to YC13-554, suggesting a potential increase of initial substrate for FA biosynthesis in YC13-559. The conversion of pyruvate to FAs involves at least 14 enzymes and/or protein complexes (**Figure 6C**). As in *Arabidopsis*, most of the individual enzymes are encoded by multiple homologous genes in *B. napus*. The pyruvate dehydrogenase complex (PDHC), catalyzing the oxidative decarboxylation of pyruvate to form acetyl-CoA, consists of three subunits, E1, E2, and E3. We found that 3 *PDHC* genes were upregulated (1.5-15 fold) while one was downregulated in YC13-559. ACCase converts acetyl-CoA into malonyl-CoA in the first committed step of FA synthesis. In YC13-559, two of the 3 identified *ACCase* genes were upregulated (1.5–1.9 fold) and one was downregulated. FAs are elongated in a series of condensation reactions with malonyl-ACP. This process is catalyzed by the FAS that comprises five components, namely MALONYL-CoA-ACP TRANSACYLASE (MCMT), 3-OXOACYL:ACP-SYNTHASE (KAS) I, KASII and KASIII, 3-OXOACYL-ACP REDUCTASE (KAR), HYDROXYACYL-ACP DEHYDRATASE (HAD) and ENOYL-ACP REDUCTASE (EAR). All of these genes, except *KASI* and *KASII*, were highly expressed in YC13-559 compared to YC13-554. FAs synthesized in the plastids are transported to the ER for TAG biosynthesis. LACS catalyze the esterification of FAs to acyl-CoAs, a key activation step that is essential for the utilization of FAs by most lipid metabolic enzymes. LACS are thus important enzymes for supplying the acyl-CoA pool for phospholipids and TAG biosynthesis (Shockey et al., 2002). Recent findings suggest that LACS are involved in lipid trafficking between ER and plastids in *Arabidopsis* (Jessen et al., 2015). Expression of seven LACS genes were 1.5–15 fold higher in YC13-559 compared to YC13-554, suggesting an increased acyl-CoA pool and trafficking rate for YC13-559.

Glycerol-3-phosphate (G-3-P) is an initial substrate for TAG biosynthesis (**Figure 6C**). GLYCEROL3-PHOSPHATE ACYLTRANSFERASE (GPAT) utilizes G-3-P and acyl-CoA as substrates to form lysophosphatidic acid (Lyso-PA). Expression of eight *GPAT* genes were significantly higher (1.5–15 fold) in YC13-559, possibly contributing to higher TAG accumulation. Acylation of Lyso-PA is catalyzed by 1-ACYL-sn-GLYCEROL-3-PHOSPHATE ACYLTRANSFERASE (LPAAT), resulting in phosphatidic acid (PA) formation. Next, PA is dephosphorylated by PHOSPHATIDATE PHOSPHATASE (PP) to form diacylglycerol (DAG). Two *LPAAT* genes were upregulated and two were downregulated in YC13-559. Four *PP* genes were downregulated while two were upregulated in YC13-559. In addition, two genes encoding PHOSPHATIDYLCHOLINE:DIACYLGLYCEROL CHOLINEPHOSPHOTRANSFERASE (PDCT), that catalyzes the transfer of phosphocholine head-groups from PC to DAG (Lu et al., 2009), were 2–4.5 fold higher in YC13-559. The final conversion of DAG into TAG is catalyzed by two enzymes: DIACYLGLYCEROL O-ACYLTRANSFERASE (DGAT) and PHOSPHOLIPID DIACYLGLYCEROL ACYLTRANSFERASE1 (PDAT). In *B. napus*, DGATs are encoded by four homologous genes of *Arabidopsis DGAT1* and *DGAT2*. In *Arabidopsis*, DGAT2 seems to be more efficient in DAG to TAG conversion than DGAT1 (Zhou et al., 2013). The expression of *DGAT2* in YC13-559 was approximately 6-fold higher compared to that of YC13-554. No significant difference in the expression of PDAT was observed in the two NILs. TAGs are stored in LDs which consist of caleosins and oleosins. In our data set, the expression of four out of ten *caleosin* genes were higher in YC13-559 relative to YC13-554. We did not observe increase in *oleosin* expression in the two NILs.

### Key Structural Genes in the Glucosinolate Biosynthetic Pathway Are Downregulated in YC13-559

Glucosinolates (GLS) are a group of sulfur-containing specialized metabolites in *Brassica* seeds that negatively affect nutritional quality. GLS and their breakdown products play roles in plant defense against pest and pathogens (Troncoso-Ponce et al., 2011). A previous study showed that overexpression of *STM* in *B*. *napus* downregulates key genes in the GLS pathway, and reduces GLS accumulation (Elhiti et al., 2012). In our data set, the expression of *STM* was higher in YC13-559 compared to YC13-554. We therefore analyzed the expression of key GLS pathway genes, including *CYTOCHROMEP45 CYP79B2* (*CYP79B2*), *METHYLTHIOALKYLMALATE SYNTHASE1* (*MAM1*), *CYTOCHROMEP45 CYP83B1* (*CYP83B1*), and *SULPHOTRANSFERASE5a* (*ST5a*), in the two NILs. The transcript levels of *CYP79B2, CYP83B1* and *ST5a* were lower in YC13-559 relative to YC13-554, suggesting that higher expression of *STM* possibly affected the GLS pathway in YC13-559. Our future efforts will include profiling of GLS accumulation in the NILs.

Glucosinolate metabolism is tightly connected to phytohormones. Auxins, in particular, indole-3-acetic acid (IAA), are essential for plant growth. Indolic GLS are derived from tryptophan (Trp) and linked to IAA biosynthesis (Bak and Feyereisen, 2001). The enzymes, CYP83B1 and CYP83B2, are important for indolic GLS and IAA biosynthesis. CYP83B2 converts Trp to indo-3-acetaldoxime (IAOx), a precursor of IAA and indole-GLS. CYP83B1 catalyzes the conversion of IAOx to *S*-alkyl thiohydroximates to regulate the flux of IAOx between IAA and indolic GLS. In *Arabidopsis*, loss-of-function of *CYP83B1* results in auxin overproduction phenotypes (Bak and Feyereisen, 2001). In our data set, the transcript levels of both *CYP83B1* and *CYP83B2* were lower in YC13-559 compared to YC13-554, indicating possible upregulation of auxin biosynthesis. To support our assumption, we analyzed the expression of auxin metabolism genes in the two NILs. Transcript level of *NITRILASE*, which is involved in IAA production, was higher in YC13-559 relative to YC13-554. Amino acid-conjugated IAA are biologically inactive, perhaps to help maintain IAA homeostasis in plants. Several IAA-amido synthetases catalyze the conjugation of amino acids to IAA (Bak and Feyereisen, 2001). In addition, IAA conjugates can be hydrolyzed by amidohydrolases, such as IAR3 and ILL2 (Rampey et al., 2004). In YC13-559, we found upregulation of *IAA-amido synthetase* and downregulation of *IAR3* (Supplementary Table S4). Our results suggest that upregulation of *amido-synthatase*, and downregulation of *amido hydrolase*, possibly play roles in maintaining optimal auxin levels during seed development. Previously, Taipalensuu et al. (1997) reported that the myrosinase-binding protein, MYROSINASE-BINDING PROTEIN 2 (MIB2), is involved in the conversion of metabolized GLS to other defense compounds (Taipalensuu et al., 1997). We found that the expression of *MIB2* was more than fourfold higher in YC13-559 compared to YC13-554.

## Conclusion

Comparative transcriptome analysis of a pair of *B. napus* NILs, YC13-554 and YC13-559 which differ significantly in seed oil accumulation revealed 1,234 candidate DEGs between the two lines that are mostly involved in lipid and carbohydrate metabolism. In addition, a number of genes associated with signal transduction, transport, and biosynthesis of phytohormones, such as JA, GA, and IAA, were found to be differentially expressed. Identification of DEGs related to phytohormones in the NILs is of particular interest. Phytohormones impose drastic phenotypes, often through interactions with signal pathway proteins that form complexes with TFs. Therefore, small changes in phytohormone metabolism can affect a large number of transcriptional cascades that regulate various biological processes. The differential expression of several lipid-related TF genes, such as *STM, LEC1, ABI3, WRI1, FUS3*, in the NILs, are potentially influenced by these phytohormones. The upregulation of these TFs in YC13-559 probably led to the increased expression of key pathway genes, including *PK, ACCase, LACS, FATA/FATB*, and *DGAT*, resulting in higher seed oil content in YC13-559. In addition, the differential expression of key TF genes is likely responsible for the downregulation of GLS pathway genes in YC13-559. It is highly possible that genetic variations affecting other pathways, e.g., that of carbohydrate metabolism, also contribute to the distinct lipid phenotypes of the NILs. Our comparative transcriptomic analysis of the *B*. *napus* NILs to elucidate DEGs that potentially affect seed oil accumulation provides a broader insight into gene networks involved in lipid biosynthesis and metabolism.

## Author Contributions

JW and CD designed research; CL, JF, JW, and CD performed experiments; SS, JW, SP and LY analyzed data; SS, LY, SP, and JW wrote the paper.

## Conflict of Interest Statement

The authors declare that the research was conducted in the absence of any commercial or financial relationships that could be construed as a potential conflict of interest.

## Footnotes

1 http://aralip.plantbiology.msu.edu

2 https://www.R-project.org

3 https://cran.r-project.org/web/packages/gplots/index.html

4 http://www.appliedbiosystems.com
