# Supplementary material for: Comparative Transcriptomic Analysis of Two Brassica napus Near-Isogenic Lines Reveals a Network of Genes That Influences Seed Oil Accumulation
Source: Front Plant Sci. 2016 Sep 29;7:1498. doi: 10.3389/fpls.2016.01498 (PMC5040705; doi:10.3389/fpls.2016.01498)
Supplement: Supplementary file 2 [file Image_1.PDF]

Supplementary Information

Comparative transcriptomic analysis of two *Brassica napus* near-isogenic lines reveals a network of genes that influences seed oil accumulation

Jingxue Wang[1*‡], Sanjay Kumar Singh[3*], Chunfang Du[2], Chen Li[1], Jianchun Fan[2], Sitakanta Pattanaik[3], and Ling Yuan[1,3‡]

[1]College of Life Sciences, Shanxi University, Taiyuan, Shanxi, 030600, China; [2]Cotton Research Institute of Shanxi Academy of Agricultural Sciences, Yuncheng, 044000, China; and [3]Department of Plant and Soil Sciences, University of Kentucky, Lexington, Kentucky 40546, U.S.A.

*These authors contributed equally to the work.
‡Correspondence:
Jingxue Wang (jingxuew@sxu.edu.cn),
Ling Yuan (lyuan3@uky.edu)

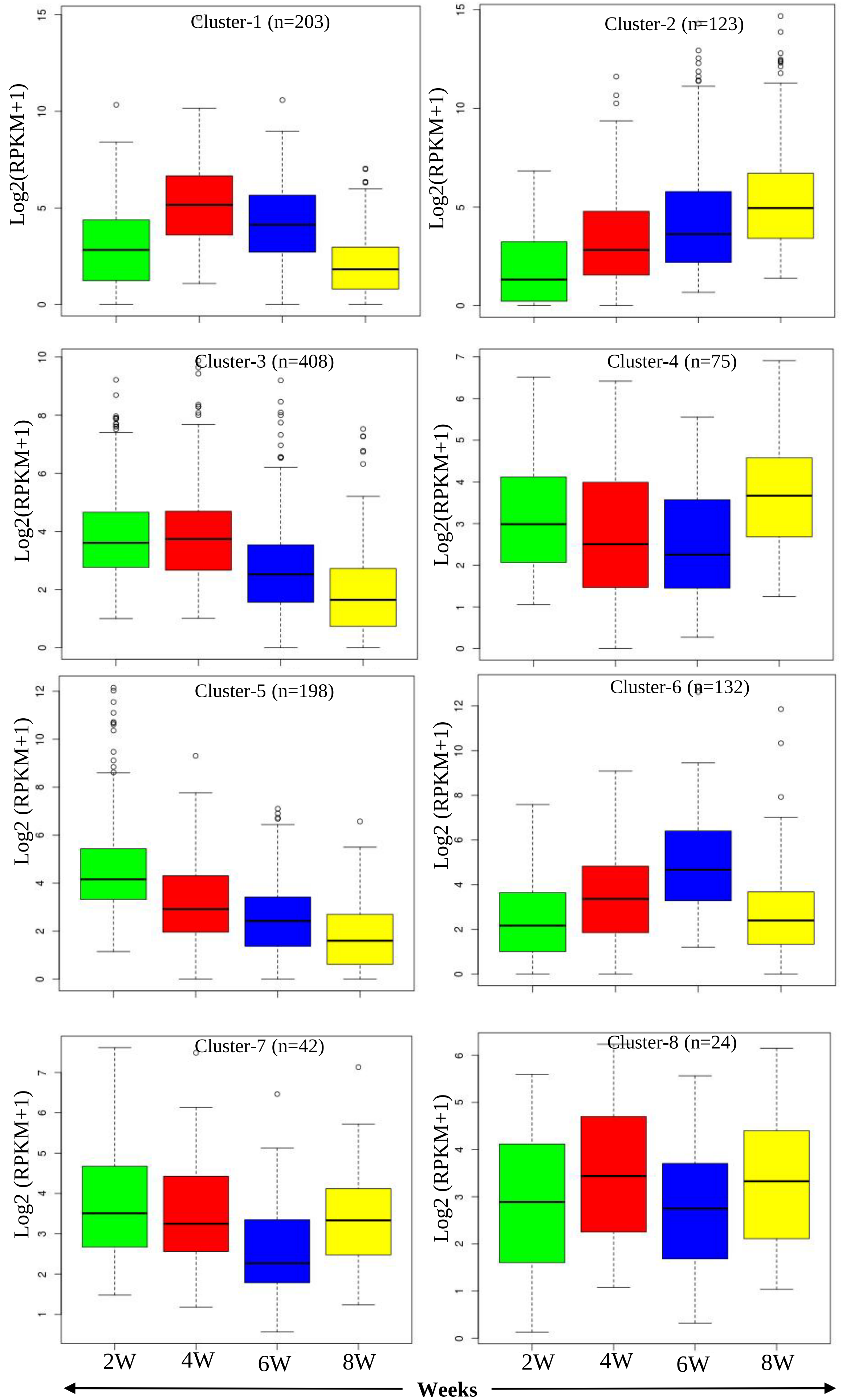

Figure S1. Expression analysis of acyl lipid-metabolism (ALM) genes during seed development in Brassica napus. Boxplot of the mean expression values [log2 (RPKM+1)] of 8 clusters of ALM genes in seeds at different developmental stages. 2W: 2 weeks; 4W: 4 weeks; 6W: 6 weeks and 8W: 8 weeks. Expression data was generated using the publically available datasets from the sequence read archive database (accession number SRP069360)

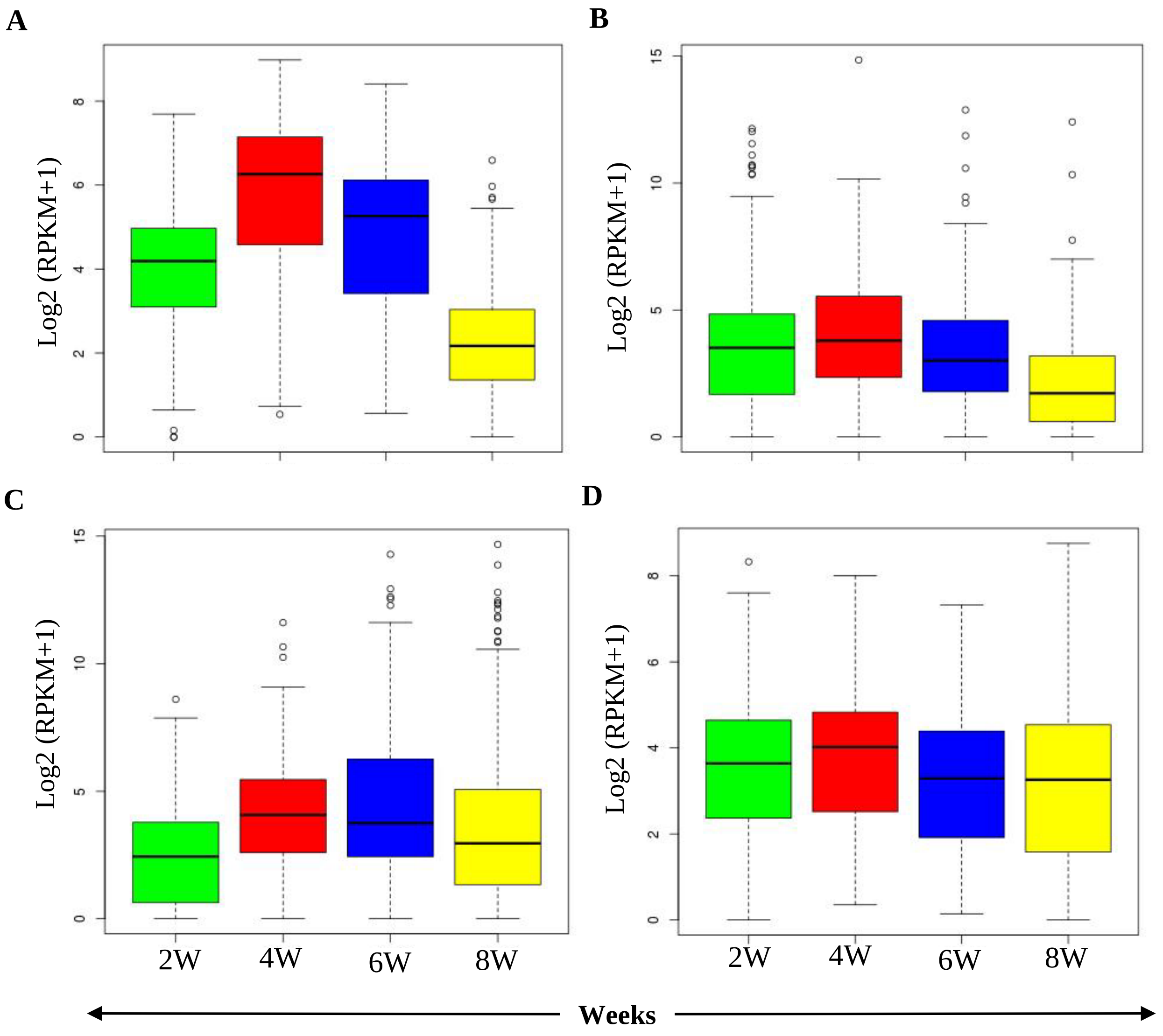

Figure S2. Boxplot of the mean gene expression values [log2 (RPKM+1)] of 4 pathways of acyl lipid-metabolism at different seed developmental stages in B. napus. (A) Fatty acid synthesis (B) Fatty acid elongation (C) Triacylglycerol biosynthesis (D) Triacylglycerol and fatty acid degradation. 2W: 2weeks; 4W: 4 weeks; 6W: 6 weeks and 8W: 8 weeks.

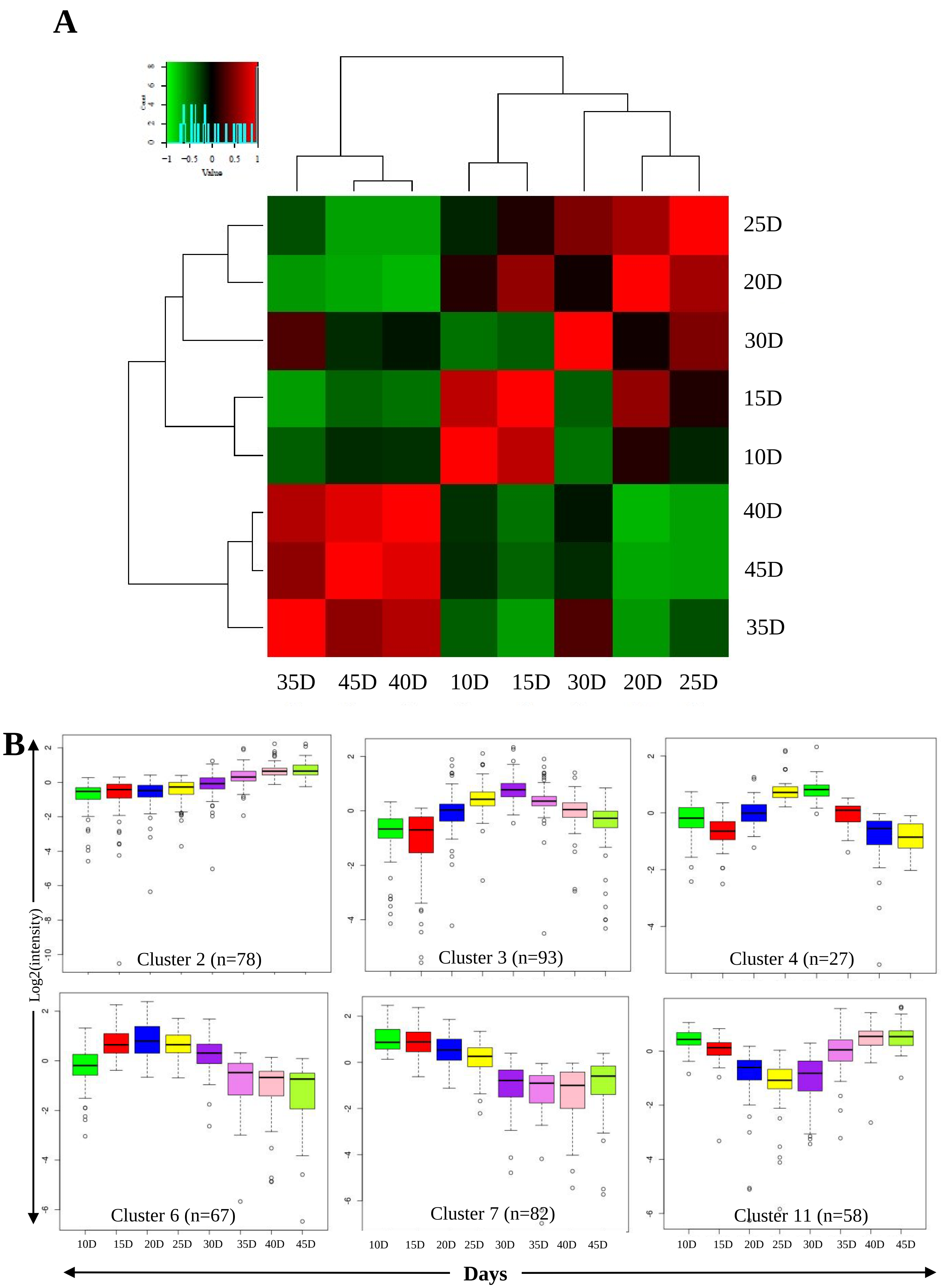

Figure S3. Microarray analysis of ALM genes during seed filling in B. napus. (A) Heat map of Spearman's correlation of the expression levels from all samples compared against each other, represented by a colored field ranging from green (-1.0) to red (1.0). (B) Boxplot of the mean expression values of 6 largest clusters of ALM genes at different seed developmental stages in B. napus. 10D:10 days; 15D:15 days; 20D:20 days; 25D:25 days; 30D:30 days; 35D:35 days; 40D:40 days; 45D:45 days.

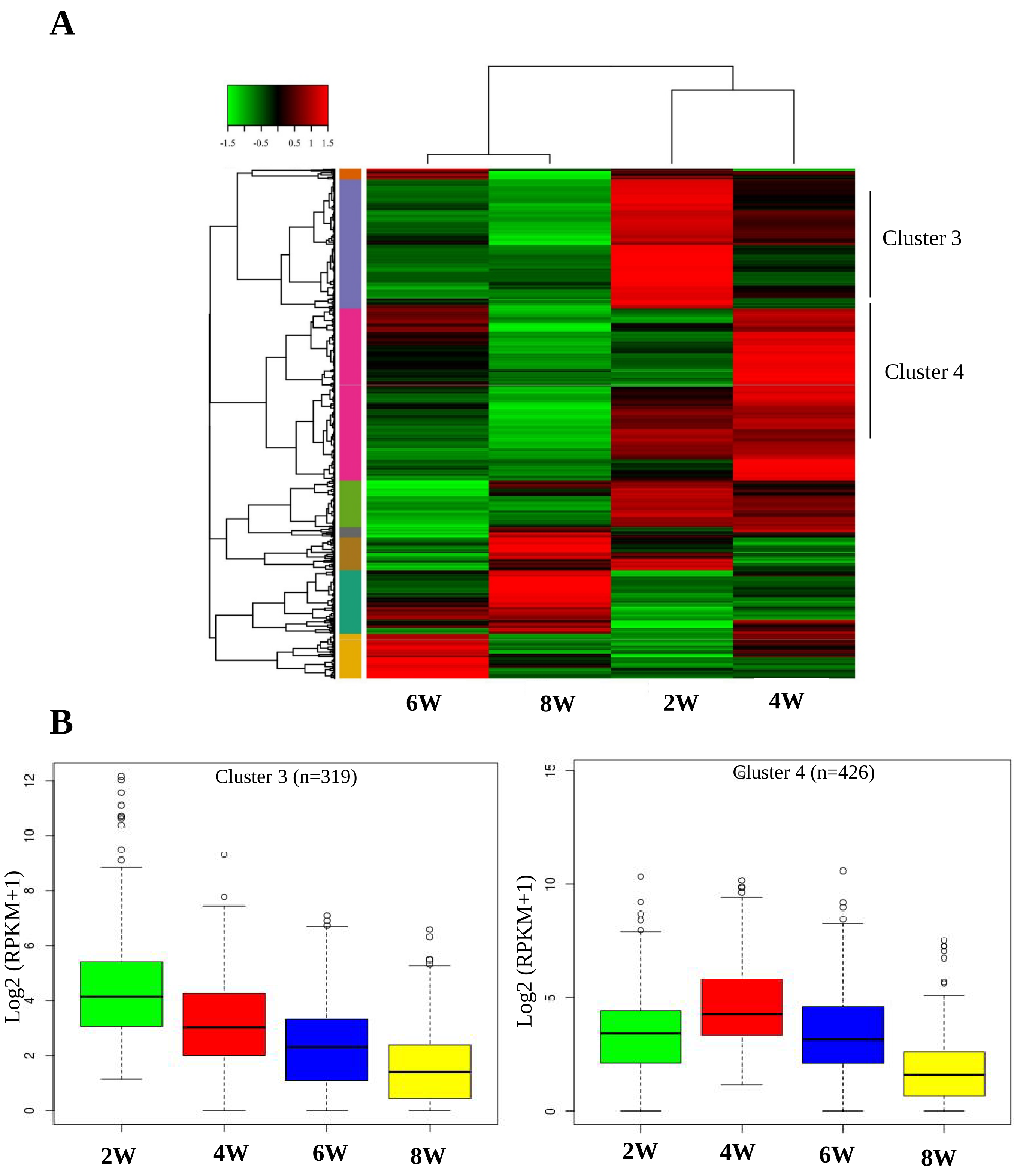

Figure S4. Cluster analysis of ALM genes and transcription factors (TFs) of "RNA" category. (A) All genes were categorized into 7 distinct clusters based on their expression pattern. Heat map shows relative expression among all groups. (B) Boxplot of the mean expression values of 2 largest clusters of ALM genes at different seed developmental stages in B. napus. 2W: 2weeks; 4W: 4 weeks; 6W: 6 weeks and 8W: 8 weeks.
